# The role of work in suicidal behavior – uncovering priorities for research and prevention

**DOI:** 10.5271/sjweh.4051

**Published:** 2022-08-31

**Authors:** Birgit A Greiner, and Ella Arensman

**Affiliations:** 1 School of Public Health, University College Cork, Ireland; 2 National Suicide Research Foundation, University College Cork, Ireland; 3 Australian Institute for Suicide Research and Prevention, School of Applied Psychology, Griffith University, Brisbane, Australia

More than 700 000 people die by suicide each year, and many more engage in suicide attempts and experience a mental disorder. Suicides particularly affect the working age population. It is the fourth leading cause for death in young people aged 15–29; more than half of global suicides (58%) in 2019 occurred before the age of 50 years (1). This topic is gaining attention in workplace settings throughout the world. The World Health Organization estimated that in a company of 1000 employees, 200–300 workers will suffer from a serious mental health problem in any given year, one worker will die by suicide every ten years, and for every employee who dies by suicide, another 10–20 will make a suicide attempt. Recent epidemiological research indicates an increasing trend in workplace related suicides (2).

There is meta-analytical evidence indicating workplace settings associated with suicide risk, especially among people working in personal services, such as the health, construction and production, and agricultural sectors. Elementary workers had the highest suicide risk, which reflects the general finding of a gradient with the lowest skilled occupations experiencing a higher risk than highly skilled workers (3).

Work-related suicide came to the attention of the wider public with the extensive media coverage of a cluster of 43 suicides in one company, France Telecom (4), and another cluster of 18 completed and attempted suicides within one year in the Chinese company, Foxconn (5). Waters et al (6) suggested that the stressful working conditions and poor management at France Telecom contributed to these tragic cases. The cluster involved cases including persons who took their lives in their workplace location, which further underlines a link between the work setting and suicide (7). The suicide cluster involving employees from France Telecom culminated in a court case and sentencing of members of France Telecom senior management (8). While the sensationalized media coverage of these two cases sparked wide criticism in relation to inappropriate mass media coverage potentially leading to copycat suicides in the respective organizations (5), the fundamental question remains: can work contribute to people taking their own lives? Is it that simple?

## The role of employment and unemployment in suicide

Historical research had pursued the issue of the work-relatedness of suicide much earlier under the notion that non-work can make people take their own lives. The French Sociologist Émile Durkheim explained suicide in the societal context rather than by solely individual factors. For example, major societal changes, such as economic recession and loss of work, can be important contributors to a so-called “anomic suicide” (9). The suicide–unemployment association has been discussed by academics since the publication of Durkheim’s classic study over 100 years ago, which concluded that unemployment increased social isolation, which then exacerbated the risk of suicide. This discussion has been rekindled by the recent COVID-related losses of jobs and economic recessions in some countries (10). Recently published systematic reviews documented increased suicide risk among the unemployed even after adjustment for pre-unemployment mental health (11,12), with a particular high risk among those in long-term unemployment (13); unemployment also increases the risk of suicide attempts and suicidal ideation (12).

Can one conclude that work is good for individuals and may even protect against suicide and mental health problems? Can work give a ‘lifeline’ and help people to stay connected to life? The answer may be that it depends on the job. Whereas employment in general is associated with a decreased risk of suicidal behavior compared to unemployment, employment in jobs associated with adverse working conditions may be a risk factor for suicidal behavior, as indicated by evidence on the association between specific psychosocial work risks and suicide.

## Psychosocial work risks associated with suicidal behavior

There has been a recent influx of methodologically sound studies, including large cohorts with a long follow-up time showing that specific psychosocial work factors are associated with a higher suicidal behavior risk. A systematic review of 22 studies demonstrated that exposure to psychosocial job stressors was associated with elevated risk of suicide ideation, attempts and death (14). Especially lack of job control and social support from supervisors and colleagues for both genders and high job demands among males has been associated with an increased suicide risk (9). Recent studies also indicated increased risks for suicide death among those exposed to sexual harassment [hazard ratio (HR) 2.82, 95% confidence interval (CI) 1.4 –5.34] (15), long working hours, ie, 45–52 weekly hours (HR 3.89, 95% CI 1.06–14.29) and >52 hours (HR 3.74, 95% CI 1.03–13.64) (16), job strain (a combination of high demands and low control) (HR 1.28, 95% CI 1.09–1.51 among men and HR 1.35, 95% CI 1.03–1.77 among women) and iso-strain (a combination of high demands, low control and low social support) (HR 1.29, 95% CI 1.09–1.53 among men and HR 1.36, 95% CI 1.04–1.78 among women) (17), workplace bullying mainly among men (HR 2.92, 95% CI 1.74–4.91) (18) and a decreased risk for those in ‘active jobs’ (high control/high demands) (HR 0.64, 95% Cl 0.60–0.70) (19). Notably, most of these recent longitudinal studies adjusted the effect measures for a range of sociodemographics, personal risk factors and baseline mental health, thereby investigating the independent contribution of working conditions associated with suicide. While some of the risks are substantially elevated, some are also imprecise estimates and based on small case numbers as death by suicide is a rare event. Results are inconclusive in relation to gender differences due to even smaller case numbers among women, as suicide is more common among men. With increasing research in this area, meta-analyses providing more precise pooled estimates of the exposure to specific psychosocial work exposures may become feasible in the future with detailed sub-group analyses.

## Theoretical challenges

While research into the role of specific job stressors on suicidal behavior can be considered in its infancy, the growing body of epidemiological evidence has the potential to scientifically establish poor psychosocial working conditions as a risk factor among the pool of other well-known risks factors for suicidal behavior. Suicidal behavior is complex and cannot be explained by one causal factor. Suicide research usually differentiates between underlying risk factors and precipitating events in the immediate environment triggering suicidal behavior. A model to explain the complexity is the Diathesis-Stress Model of suicide. It defines stressors as the recurrence of depressed mood or psychotic symptoms within the course of a psychiatric disorder or a negative stressful life event, such as losing one’s job or the death of family member or friend. Such a stressor may lead to the subjective experience of depressed mood and suicidal thoughts, which may induce suicidal ideation and increase the risk of a suicidal crisis via interaction with a trait disposition, such as hopelessness and neuroticism (20, 21).

The addition of specific occupational characteristics to the canon of preconditions is relatively novel and requires the expansion of theoretical frameworks to explain the mechanisms that link workplace factors among other contributors to suicidal behavior. In suicide research, workplace factors have been generally conceptualized as factors immediately precipitating suicidal behavior, eg, in the context of job loss, or traumatic work events such as violence (20, 22). The theoretical integration of chronic exposure to work stressors as underlying risk factor with their accumulative impact on vulnerability and onset / aggravation of mental health problems would create an opportunity to integrate the scientific evidence of two academic disciplines: psychiatry and occupational epidemiology. The first models have now been published with integration of the “interpersonal-psychological theory of suicide” and the “psychache theory of suicide” with integration of work factors (23). The framework specifies work-related predictors (eg, unemployment, unstable employment, job demands, relationships at work, burnout, coping styles among others), direct antecedents (eg, entrapment, thwarted belonginess), and further contributors (physical work environment) and mechanisms (acclimatization to pain, acquired capability for suicide) (23).

## Implications for future research

Although essential in specifying risk factors, epidemiological studies usually do not explain the complex mechanisms that link adverse working conditions to suicide. For example, it is not clear how the psychological effects of the exposure to chronic adverse working conditions or critical events at work accumulate over time to reach a crisis point and develop into mental health conditions and suicidal behavior. This is where more longitudinal trajectory studies are needed. Scarce research identified specific “low-quality employment trajectories” (24) for poor mental health. Other research specifically examined the transitions between exposure patterns of adverse working conditions, onset of a mental disorder, suicide attempts and completed suicide in the context of long working hours (16), one study showed distinct trajectories of work-related functional impairment after an occupational accident or injury among individuals with subsequent suicide (25).

Psychological autopsy studies are needed to clarify the complexity of interrelated factors and create an in-depth understanding of their interactions as well as obtaining insights into protective factors via large-scale nationwide studies using a case–control design and matching suicide cases with controls (26). Psychological autopsy is an in-depth research tool used to examine suicide, which involves collecting all available information on the deceased via structured interviews with family members, relatives or friends as well as obtaining information from coroners and healthcare professionals resulting in quantitative and qualitative data (27).

There is little evidence on the role of protective work characteristics for suicidal behavior except for the protective nature of employment itself. Stress research suggests that well-managed work can offer valuable external coping resources for individuals to actively deal with stress and develop and maintain their coping skills and ultimately their mental health and wellbeing (28). Protective work characteristics include eg, job control, autonomy, opportunities to learn, peer support, supervisor support, opportunities for professional development, and constructive feedback (28, 29). More research is needed to determine whether those work characteristics also play a protective role for suicidal behavior. Such research may be built on the published evidence synthesis of risk factors and protective and resilience building work factors for nurses (30) and the police force (31), and research based on expansions of the demand–control model to include the mechanism of the development of coping in the context of working conditions (32). Identifying protective factors would be important to develop guidelines how organizations can provide a ‘lifeline’ by creating supportive structures and fulfilling and engaging jobs.

## Implications for policy and practice

While some scholars suggest that improving access to evidence-based interventions for minor stress-related depressive symptoms in occupational sectors associated with high suicide rates is likely to prevent the development of severe depressive disorders and comorbidities, and subsequent suicidal behavior there are only few workplace suicide prevention initiatives that have been systematically evaluated for effectiveness (33, 34). However, the scarce available evidence indicates that prevention initiatives can have beneficial effects, including workplace suicide prevention policies, stigma reduction campaigns, educational and gatekeeper training, and suicide bereavement support resources. It is suggested that the simultaneous implementation of multiple interventions is likely to result in greater reductions of suicide and non-fatal suicidal behavior (34). In this regard, an example of a multi-component suicide prevention program in the workplace is Mates in Construction (MATES), initiated in Queensland, Australia. MATES uses onsite universal psychoeducation to encourage help-seeking and early intervention through ‘connectors’ trained in suicide first aid and supported by outreach, case management, a 24-hour telephone response line, and online counselling. A cost-effectiveness analysis demonstrated that MATES could potentially avert 0.4 suicides, 1.01 work disability cases, and 4.92 short-term absences (35).

With the recognition of a poor psychosocial work environment as a risk factor for suicidal behavior, the workplace not only emerges as a relevant setting for suicide prevention and mental health promotion, but also psychosocial working conditions themselves become of interest as modifiable determinants of mental health and suicidal behavior (36, 37). There is a lack of interventions with upstream primary-level approaches to improve the quality of psychosocial working conditions in an effort to prevent mental health problems and suicidal behavior that complement secondary and tertiary-level workplace interventions (33, 36). Increased attention has especially been placed on multi-level workplace interventions that target changes in the entire organization (eg, workplace modifications to reduce stressors and enhance resources, mental health policies and cultural changes in relation to open discussion about mental health), among leaders/managers (eg, supervisor awareness training and skills training to handle mental health issues and to create healthy working conditions), in groups and teams (eg, peer support systems), and individual workers (increasing knowledge and resilience through education and training) with a range of promising projects underway, including large-scale EU Horizon 2020 funded projects such as MENTUPP and H-Work (38, 39). While the COVID-19 pandemic has contributed to increased mental health challenges for some populations and workplace settings, in particular among people with pre-existing mental health conditions, proactive and innovative mental health interventions are required in the workplace, including digital intervention and prevention programs (38, 40).

Suicide prevention has the potential to be integrated into existing workplace mental health activities and work systems. In addition, increased productivity and associated economic gains have been demonstrated for workplace mental health interventions (33). To date, most research in this area has been carried out in high income countries and research on work-related suicides in low- and middle-income countries is needed. Notwithstanding the research gaps and methodological limitations, the role of work in suicidal behavior requires greater prioritization within national suicide prevention policies and interdisciplinary capacity building for it to be fully uncovered.

## References

[1] World Health Organisation (WHO) Suicide worldwide in 2019.

[2] Peterson C, Sussell A Rates by Industry and Occupation - National Violent Death Reporting System, 32 States,2016 [Internet]. MMWR Morb Mortal Wkly Rep 2020.

[3] Milner A, Spittal MJ, Pirkis J, LaMontagne AD (2013). Suicide by occupation:systematic review and meta-analysis. Br J Psychiatry.

[4] Bossard C, Santin G, Lopez V, Imbernon E, Cohidon C (2016). Surveillance of work-related suicide in France:an exploratory study. Rev Epidemiol Sante Publique.

[5] Cheng Q, Chen F, Yip PS (2011). The foxconn suicides and their media prominence:is the Werther Effect applicable in China?. BMC Public Health.

[6] Waters S, Karanikolos M, McKee M (2016). When work kills. J Public Mental Health.

[7] Skogstad M, Skorstad M, Lie A, Conradi HS, Heir T, Weisæth L (2013). Work-related post-traumatic stress disorder. Occup Med.

[8] BBCNews (2019). France Télécom suicides:Three former bosses jailed.

[9] Durckheim E (1897). Le suicide - Etude de sociologie [Suicide - a sociology study. Paris:Ancienne Librerie Germer Bailliere.

[10] Bastiampillai T, Allison S, Looi JC, Licinio J, Wong ML, Perry SW (2020). The COVID-19 pandemic and epidemiologic insights from recession-related suicide mortality. Mol Psychiatry.

[11] Milner A, Page A, LaMontagne AD (2014). Cause and effect in studies on unemployment, mental health and suicide:a meta-analytic and conceptual review. Psychol Med.

[12] Amiri S (2020). Unemployment and suicide mortality, suicide attempts, and suicide ideation:A meta-analysis. Int J Mental Health.

[13] Milner A, Page A, LaMontagne AD (2013). Long-term unemployment and suicide:a systematic review and meta-analysis. PloS One.

[14] Milner A, Witt K, LaMontagne AD, Niedhammer I (2018). Psychosocial job stressors and suicidality:a meta-analysis and systematic review. Occup Environ Med.

[15] Hanson LLM, Nyberg A, Mittendorfer-Rutz E, Bondestam F, Madsen IE (2020). Work related sexual harassment and risk of suicide and suicide attempts:prospective cohort study. BMJ.

[16] Lee HE, Kim I, Kim HR, Kawachi I (2020). Association of long working hours with accidents and suicide mortality in Korea. Scand J Work Environ Health.

[17] Niedhammer I, Chastang JF, Coutrot T, Geoffroy-Perez B, Lamontagne d A, Milner A (2020). Psychosocial work exposures of the job strain model and suicide in France:findings from the STRESSJEM prospective study of 1.5 million men and women over 26 years of follow-up. Psychother Psychosom.

[18] Conway PM, Erlangsen A, Grynderup MB, Clausen T, Rugulies R, Bjørner JB (2022). Workplace Bullying and Risk of Suicide and Suicide Attempts:A Register-Based Prospective Cohort Study of 98 330 Participants in Denmark. Scand J Work Environ Health.

[19] Åberg M, Staats E, Robertson J, Schiöler L, Torén K, LaMontagne AD (2022). Psychosocial job stressors and risk of suicidal behavior - an observational study among Swedish men. Scand J Work Environ Health.

[20] Hawton K, van Heeringen K (2009). Background and epidemiology. Lancet.

[21] Brodsky BS, Mann JJ (2017). Suicide* In:Reference Module in Neuroscience and Biobehavioral Psychology [Internet]. Elsevier.

[22] Hawton K, Agerbo E, Simkin S, Platt B, Mellanby RJ (2011). Risk of suicide in medical and related occupational groups:a national study based on Danish case population-based registers. J Affect Disord.

[23] Howard MC, Follmer KB, Smith MB, Tucker RP, Van Zandt EC (2022). Work and suicide:An interdisciplinary systematic literature review. J Organ Behav.

[24] Da Silva MA, Younès N, Leroyer A, Plancke L, Lemogne C, Goldberg M (2019). Long-term occupational trajectories and suicide. Scand J Work Environ Health.

[25] Wang M, Björkenstam C, Alexanderson K, Runeson B, Tinghög P, Mittendorfer-Rutz E (2015). Trajectories of work-related functional impairment prior to suicide. PloS One.

[26] Kodaka M, Matsumoto T, Takai M, Yamauchi T, Kawamoto S, Kikuchi M (2017). Exploring suicide risk factors among Japanese individuals:The largest case-control psychological autopsy study in Japan. Asian J Psychiatry.

[27] Arensman E, Larkin C, McCarthy J, Leitao S, Corcoran P, Williamson E (2019). Psychosocial, psychiatric and work-related risk factors associated with suicide in Ireland:optimised methodological approach of a case-control psychological autopsy study. BMC Psychiatry.

[28] Nielsen K, Nielsen MB, Ogbonnaya C, Känsälä M, Saari E, Isaksson K (2017). Workplace resources to improve both employee well-being and performance:A systematic review and meta-analysis. Work Stress.

[29] Bakker AB, Demerouti E (2007). The Job Demands-Resources model:state of the art. J Manag Psych.

[30] Yu F, Raphael D, Mackay L, Smith M, King A (2019). Personal and work-related factors associated with nurse resilience:A systematic review. Int J Nurs Stud.

[31] Chae MH, Boyle DJ (2013). Police suicide:Prevalence, risk, and protective factors. Polic Int J Police Strateg Manag.

[32] Wallace JE (2017). Burnout, coping and suicidal ideation:an application and extension of the job demand-control-support model. J Workplace Behav Health.

[33] Milner A, Page K, Spencer-Thomas S, LaMontagne AD (2015). Workplace suicide prevention:a systematic review of published and unpublished activities. Health Promot Int.

[34] Witt K, Milner A, Allisey A, Davenport L, LaMontagne AD (2017). Effectiveness of suicide prevention programs for emergency and protective services employees:A systematic review and meta-analysis. Am J Ind Med.

[35] Doran CM, Ling R, Gullestrup J, Swannell S, Milner A (2016). The impact of a suicide prevention strategy on reducing the economic cost of suicide in the New South Wales construction industry. Crisis J Crisis Interv Suicide Prev.

[36] LaMontagne AD, Martin A, Page KM, Reavley NJ, Noblet AJ, Milner AJ, In Hudson HL, Nigam, Sauter SL, Chosewood LC, Schill AL, Howard J (2019). Developing an integrated approach to workplace mental health. Total worker health MA.

[37] LaMontagne AD, Keegel T, Louie AM, Ostry A (2010). Job stress as a preventable upstream determinant of common mental disorders:A review for practitioners and policy-makers. Adv Ment Health.

[38] Arensman E, O'Connor C, Leduc C, Griffin E, Cully G, NíDhálaigh D (2022). Mental Health Promotion and Intervention in Occupational Settings:Protocol for a Pilot Study of the MENTUPP Intervention. Int J Environ Res Public Health.

[39] De Angelis M, Giusino D, Nielsen K, Aboagye E, Christensen M, Innstrand ST (2020). H-WORK Project:Multilevel Interventions to Promote Mental Health in SMEs and Public Workplaces. Int J Environ Res Public Health.

[40] Giusino D, De Angelis M, Pietrantoni L, Black NL, Neumann WP, Noy I (2021). Digital Solutions for Workplace Mental Health Promotion During COVID-19 Pandemic:Taxonomy and Human Factors Issues. Proceedings of the 21st Congress of the International Ergonomics Association (IEA 2021).

